# Tait’s Operation in Texas, Letter from Dr. Baldwin of Ohio

**Published:** 1886-11

**Authors:** 


### "TAIT'S OPERATION (?) IN TEXAS."

### NOTE FROM DR. BALDWIN, OF OHIO.

*Editor of Daniel's Texas Medical Journal:*

Whether Tait's operation has been performed five times in Texas or fifty, may depend upon what constitutes a Tait operation. Of the cases reported by Dr. Cupples, as given in your October issue, pages 166–7, only one—case 3—is a passable Tait, and that is too briefly reported to be certain. You say Dr. Hadra had three Tait's, and Dr. Christian one; but the conditions found at these operations are not given.

In a letter from Dr. Battey, dated October 1, '86, he defines his operation as follows:

"The complete extirpation of both ovaries whilst yet in a state of functional activity, for the effectual remedy of cases of disease, otherwise incurable." Under date of October 8, '86, Mr. Tait writes me: "I mean by Tait's operation the removal of uterine appendages for disease, actual physical disease, other than cystoma. That is to say, an operation for removal of all the uterine appendages, both ovaries and tubes, for myoma of the uterus, is a Tait operation. Removal of one tube for pyosalpinx, or other disease, is a Tait operation. Removal of both ovaries and tubes for chronic inflammatory disease and adhesions, is a Tait operation. Removal of appendages which are not diseased, and are not removed on account of disease of the uterus, is not a Tait operation. The latter is an operation of which I do not approve, because the results have not been satisfactory."

What is meant by a Battey operation, is pretty well understood in this country; and it is quite generally supposed that a Tait operation is simply a Battey, plus removal of the tubes; but such a definition is incorrect.

